# Early Diagnosis of V180I Genetic Creutzfeldt-Jakob Disease at the Preserved Cognitive Function Stage

**DOI:** 10.7759/cureus.23374

**Published:** 2022-03-21

**Authors:** Yutaro Suzuki, Atsuhiko Sugiyama, Mayumi Muto, Katsuya Satoh, Tetsuyuki Kitamoto, Satoshi Kuwabara

**Affiliations:** 1 Department of Neurology, Chiba University Graduate School of Medicine, Chiba, JPN; 2 Department of Neurology, Chiba Rosai Hospital, Chiba, JPN; 3 Department of Health Sciences, Unit of Medical and Dental Sciences, Nagasaki University Graduate School of Biomedical Sciences, Nagasaki, JPN; 4 Department of Neurological Sciences, Tohoku University Graduate School of Medicine, Miyagi, JPN

**Keywords:** v180i, diagnosis, cognitive reserve, magnetic resonance imaging, creutzfeldt–jakob disease

## Abstract

We herein report a case of genetic Creutzfeldt-Jakob disease (CJD) due to V180I mutation in the prion protein (PrP) gene diagnosed at a preserved cognitive function stage. Although neuropsychological tests revealed normal cognitive functions, increased signal intensity in the cerebral cortices with swelling on diffusion-weighted imaging (DWI) in magnetic resonance imaging (MRI) prompted genetic testing for the PrP gene. This case suggests that cortical hyperintensity on DWI with swelling may be a useful finding of brain MRI for the diagnosis of V180I genetic CJD even at an extremely early stage, such as at the preserved cognitive function stage.

## Introduction

Prion diseases are fatal transmissible spongiform encephalopathy caused by the prion protein (PrP) gene and are classified into sporadic, genetic, and acquired forms [1]. The genetic form is a prion disease with causative mutations in the human PrP gene, including genetic Creutzfeldt-Jakob disease (CJD), Gerstmann-Sträussler-Scheinker disease, and fatal familial insomnia [2]. The most common genetic CJD in Japan is caused by a causative point mutation of valine and isoleucine at codon 180 (V180I) in the PrP [3].

The following clinical features of V180I genetic CJD are relatively uniform but significantly different from those of typical sporadic CJD [3-5]: older age onset, prolonged disease duration with a slower progression, and a lower positive rate of periodic sharp wave complexes on electroencephalography. Furthermore, patients with V180I genetic CJD rarely have a family history [3,5]. Therefore, it is difficult to diagnose at the early stage merely based on clinical features and is often misdiagnosed as other causes of dementia, such as Alzheimer’s disease [6]. Conversely, hyperintensities along the cortices on diffusion-weighted imaging (DWI) of brain magnetic resonance imaging (MRI) are also observed in patients with V180I genetic CJD with high sensitivity than those with sporadic CJD [5]. Moreover, cortical swelling at the site of DWI signal abnormalities has been reported to be a characteristic finding of V180I genetic CJD [4,7].

We herein report a patient with V180I genetic CJD diagnosed at the preserved cognitive functioning stage with brain MRI findings being key to diagnosis.

## Case presentation

**Figure 1:**
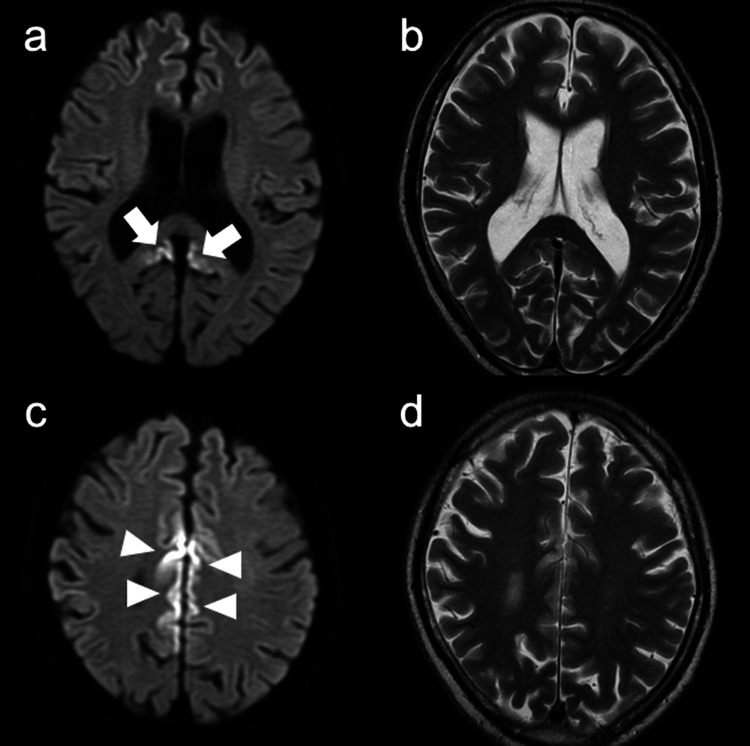
Brain magnetic resonance imaging (MRI) findings at two months before referral. Brain MRI reveals increased signal intensity in the cerebral cortices with swelling on diffusion-weighted images (A, C) and T2-weighted images (B, D) (arrows and arrowheads).

In October 2020, a 75-year-old man was referred to our department for a one-year history of mild forgetfulness and abnormal brain MRI findings. He had 16 years of education and worked as the president of a service company. Prior to his referral to our department, neuropsychological tests and brain MRI had been performed at another hospital. His Mini-Mental State Examination (MMSE) and Frontal Assessment Battery (FAB) scores were 30 out of 30 and 18 out of 18, respectively, at six months before referral to our department. The Wechsler Adult Intelligence Scale-III (WAIS-III) indicated a verbal intelligence quotient (IQ) of 147, a performance IQ of 131, and a full-scale IQ of 142. At two months before referral, his DWI and T2-weighted imaging in MRI showed increased signal intensity with swelling in the cerebral cortices except for the medial occipital and cerebellar cortices (Figure 1).

His wife reported that he would forget where he put things and repeat the same questions. His daily life and professional activities as a company president were preserved. Past medical history was unremarkable, except for appendicitis. No family history of dementia was reported. Neurological examination was unremarkable. He refused to undergo further investigations at that time.

**Figure 2:**
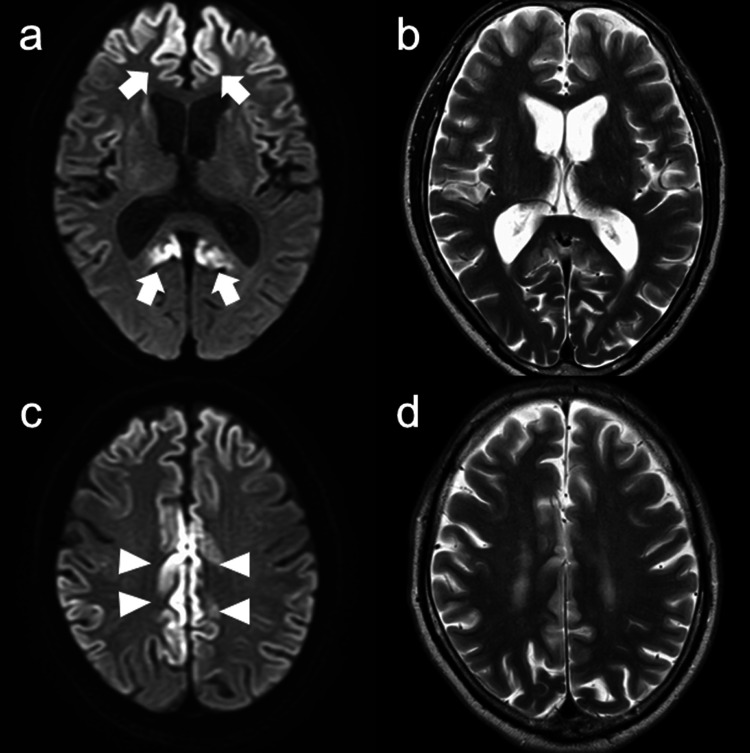
Brain magnetic resonance imaging (MRI) findings at diagnosis. Brain MRI reveals extension of abnormal signals along the cerebral cortices with swelling on diffusion-weighted images (A, C) and T2-weighted images (B, D).

In January 2021, the patient was re-examined due to worsening forgetfulness. His neurological examination was normal. The MMSE, FAB, and Addenbrooke’s Cognitive Examination III (ACE-III) scores [8] were 28, 17, and 90, respectively. The Wechsler Memory Scale-Revised version revealed that his verbal memory was 101, visual memory was 114, general memory was 106, attention was 112, and delayed recall was 86. Electroencephalography showed no obvious abnormalities. No pleocytosis was observed in the CSF. The total tau protein in the CSF was elevated (1631 pg/ml, cutoff < 1300), and the 14-3-3 protein was detected by western blotting. Results of the real-time quaking-induced conversion (RT-QuIC) assay were negative. Brain MRI revealed the extension of abnormal signals along the cerebral cortices (Figure 2). The PrP analysis revealed V180I mutation. The polymorphic codon 129 indicates methionine/valine (M/V) heterozygosity and codon 219 indicates glutamate homozygosity.

The intellectual performance of the patient deteriorated progressively. In June 2021, his cognitive function had decreased to MMSE 23, FAB 7, and ACE-III 66. Myoclonus and cerebellar ataxia were also observed. In September 2021 (23 months after the disease onset), the patient reached the akinetic mutism state.

## Discussion

The patient was diagnosed with V180I genetic CJD at the preserved cognitive function stage with MMSE 29, FAB 17, and ACE-III 90. Given the development of new potential therapies to alter the natural history of prion diseases and inclusion in clinical trials before the occurrence of irreversible neurodegeneration, early diagnosis, especially in preserved cognitive function stage, has important clinical implications [9,10].

There are three points that seem to be important in the diagnosis of this case at the preserved cognitive function stage. First, cortical hyperintensity on DWI of brain MRI is a highly sensitive diagnostic marker for CJD, including V180I genetic CJD. A case of V180I genetic CJD with cortical hyperintensity on DWI three months before the onset of the disease has been reported [11]. Cases of sporadic CJD with DWI signal abnormalities detected at ≥ one year before the onset of neurological symptoms have also been previously reported [12-14]. Especially, cortical swelling at the site of DWI signal abnormalities has been reported as a characteristic finding of V180I genetic CJD [4,7]. In our case, cortical hyperintensity on DWI with swelling was observed at an early stage with preserved cognitive function. Therefore, cortical hyperintensity on DWI with swelling, as in the current case, should prompt genetic PrP analysis, even if the cognitive function is preserved. Second, patients with V180I genetic CJD show slower disease progression than those with sporadic CJD [3-5]. Finally, “cognitive reserve” in this case may delay the progression from the preserved cognitive function stage to dementia. Cognitive reserve is a theoretical concept that proposes greater lifetime engagement in cognitively stimulating activities, such as education and occupational attainment, which modifies the brain by reducing the negative effect of brain pathology on cognition [15]. The patient in this case report had 16 years of education, worked as a company president, and thrived in a very superior range results on the previous WAIS-III, suggesting a high cognitive reserve.

In patients with V180I genetic CJD, M/V heterozygosity at codon 129 may affect the appearance of MRI abnormalities from an early disease stage. The present case had M/V heterozygosity at codon 129. A previous case of V180I genetic CJD in which MRI abnormalities preceded the clinical onset also had 129M/V heterozygotes [11]. A close relationship between PrP polymorphisms and susceptibility to prion diseases has long been recognized [16,17]. The frequency of 129M/V heterozygotes in patients with V180I genetic CJD was approximately threefold higher than that in the general Japanese population, suggesting that 129M/V heterozygotes predispose to V180I genetic CJD [18]. In addition to the effects on susceptibility, PrP polymorphisms can affect the disease duration, neuropathology, or type of abnormal misfolded isoform of PrP [19-21]. In a report analyzing the clinical features of V180I genetic CJD in a Japanese cohort, no difference was observed in disease progression between patients with 129MM homozygotes and 129M/V heterozygotes; however, the frequency of positive CSF biomarkers, such as 14-3-3 protein, total tau protein, and RT-QuIC, was lower in patients with 129M/V heterozygotes [5]. Further studies are needed to confirm the association between codon 129 polymorphism in PrP and the timing of the appearance of MRI abnormalities in patients with V180I genetic CJD.

## Conclusions

Cortical hyperintensity on DWI with swelling is a characteristic finding of brain MRI in V180I genetic CJD and can be a key to diagnosing V180I genetic CJD, even at an early stage, such as the preserved cognitive function stage. Given the development of new potential therapies to alter the natural history of prion diseases in the future, early diagnosis, especially in preserved cognitive function stage, has important clinical implications.
